# Investigating the rheological behavior of a hybrid nanofluid (HNF) to present to the industry

**DOI:** 10.1016/j.heliyon.2022.e11561

**Published:** 2022-11-12

**Authors:** Mohammad Hemmat Esfe, Davood Toghraie, Soheyl Alidoust, Fatemeh Amoozadkhalili, Erfan Mohammadnejad Ardeshiri

**Affiliations:** a Department of Mechanical Engineering, Imam Hossein University, Tehran, Iran; b Department of Mechanical Engineering, Khomeinishahr Branch, Islamic Azad University, Khomeinishahr, Iran; c School of Chemistry, Damghan University, Damghan 36716-41167, Iran; d Nanofliuds Advance Research Group, Iran

**Keywords:** Hybrid nanofluid, Viscosity, Rheological behavior, RSM, MWCNTs, Thermophysical properties, Experimental, Statistical, Numerical, MgO

## Abstract

Hybrid nanofluids (HNFs) are potential fluids that have higher thermophysical properties than conventional nanofluids of heat transfer and viscosity. HNF is a new generation of nanofluid that is produced by dispersing two or more types of dissimilar nanoparticles (NPs) in the base fluid. In this study, the rheological behavior of MWCNT (25%)-MgO (75%)/SAE40 HNF was investigated experimentally, statistically and numerically. Temperature conditions are in the range of T = 50-25 °C, solid volume fractions (SVFs) are in the range of SVF = 0.0625–1% and shear rate (SR) is in the range of SR = 666.5–7998 s^−1^. This study aims to identify the rheological behavior of HNF based on the effective factors of temperature, SR, and SVF. Various methods show that HNFs exhibit non-Newtonian behavior. The numerical values of the power-law index (n) at T = 50 °C and SVF = 0.75% show the strongest non-Newtonian behavior of HNF and n = 0.9233 is reported. Using laboratory findings, the maximum and minimum viscosities of the base oil increase and decrease by 24% and -8.50%, respectively. Using the response surface methodology (RSM), the relationship between experimental data and modeled data is determined. A quadratic three-variable model with R^2^ = 0.9994 is used to predict the data.

## 1 Introduction

**Figure 1:**
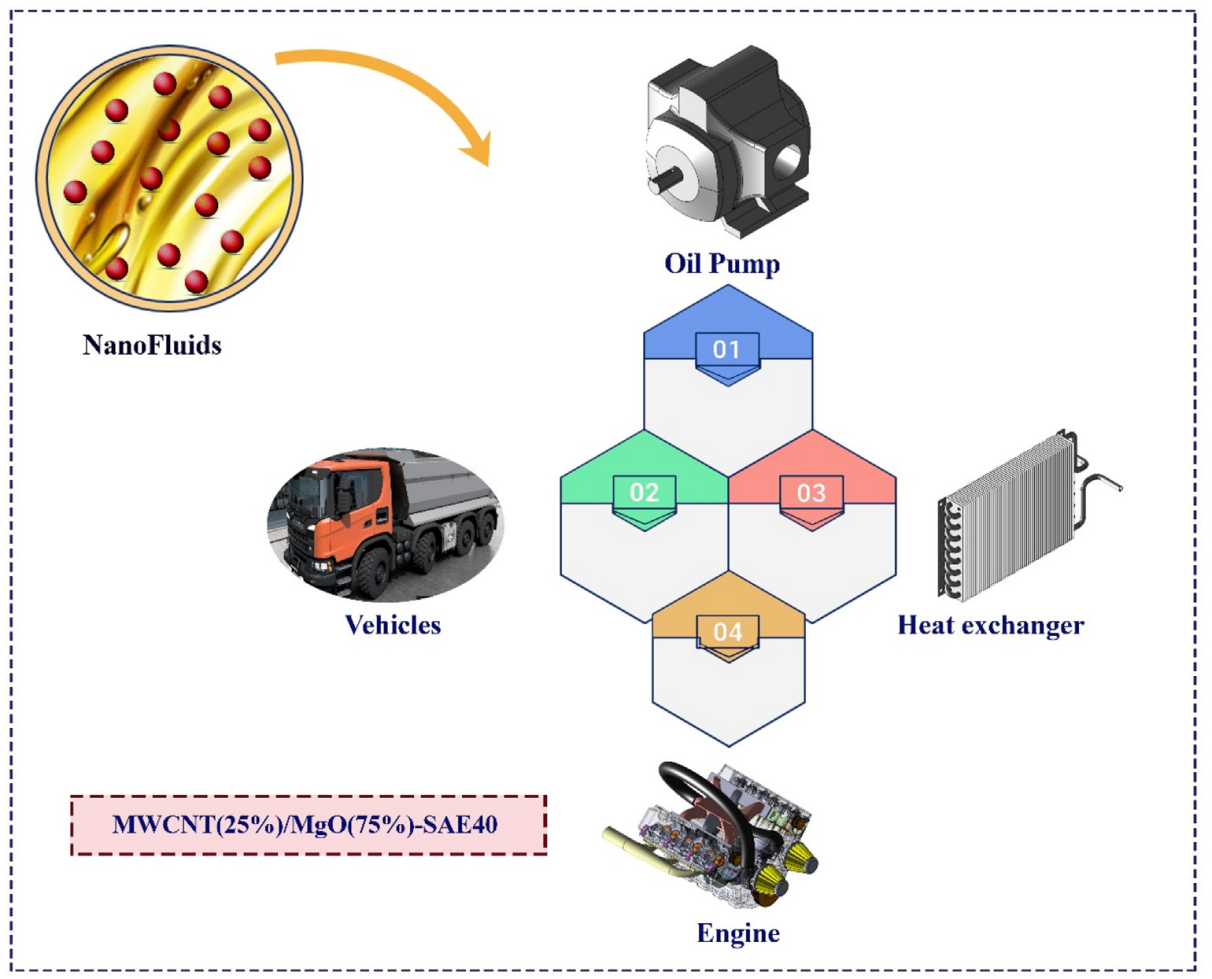
Some applications of nanotechnology in different parts of the industry.

The role of nanotechnology in the recent advances in science is very significant and this has led scientists and researchers in different fields to focus on the use of nanotechnology to improve various systems [1, 2, 3, 4, 5, 6, 7, 8, 9, 10]. One of the things that is done to improve the properties of different materials is to add additives to the structure of materials in different phases to improve their properties [11, 12, 13, 14, 15, 16]. Nanofluids (NFs) are obtained by suspending nanoparticles (NPs) in base fluids such as water, ethylene glycol (EG), propylene glycol, oil and various fluids. NFs are the evolution of fluid science. NPs have unique mechanical, thermal, magnetic and electrical properties. Many high-tech industries often face cooling technical challenges. Conventional methods such as wide surfaces and microchannels that increase the rate of heat transfer have the disadvantage of increasing the pumping power of the coolant. In recent decades, many studies were conducted to study the thermophysical properties of NFs such as viscosity (μ_nf_) [17, 18], thermal conductivity (TC) [19, 20] and convective heat transfer coefficient [21, 22]. NFs are used in various fields such as air conditioning [23], solar cell [24], automobile [25], nuclear reactors [26], lubrication [27], electrical systems [28], heat exchangers [29]. The TC of solids is higher than that of ordinary fluids, and therefore the dispersion of fine particles is expected to improve the TC of fluids. Many researchers have studied various aspecet of heat transfer and fluid flow properties of NFs in the past 15 years [30, 31, 32] and found that the heat transfer coefficients are increased by adding NPs to the base fluid. However, the enhanced thermal properties of these new fluids should not be offset by additional pumping power. Therefore, it is necessary to investigate the rheological behavior of NFs. Rheological behavior is an important parameter in fluid flow systems. To calculate the required pumping power, the rheological behavior of the fluid is required. Viscosity is one of the important parameters in liquids, which plays an important role in calculating Reynolds number, Prandtl number and heat transfer coefficient. Studies show that various parameters such as solid volume fraction (SVF), particle size, temperature, properties of the base fluid and surfactants affect the TC and μ_nf_. Several studies have been conducted on the μ_nf_ of different NFs [33, 34, 35]. Abdullahi et al. [36] reported the changes in rheological behavior and lubrication of CuO–MWCNTs/SAE40 engine oil HNF. Viscosity changes were measured in SVF = 0.0625 and 1 % and T = 25–50 °C and different SRs, the results show that at SVF = 1%, the viscosity of the HNF was 29.47% higher than the viscosity of the base oil. A new relationship was proposed in terms of SVF for each temperature, which estimates the sensitivity of HNF to a 10% increase in SVF. Alidoust et al. [37] studied thermal conductivity of SWCNT (15%)-Fe_3_O_4_ (85%)/water HNF. The initial TC increase of the produced HNF compared to water at SVF = 0.03% and T = 30 °C is 0.9%, but the maximum RTC value of 32.20% is reported, which is a significant value. All their studies about viscosity show that the *μ*_*nf*_ is higher than *μ*_*bf*_ and increases with the increase of SVF. various base fluids were utilized for all of these studies, but they were all Newtonian fluids. According to the reports in the articles, the dispersion of NPs in Newtonian base fluids has led to the fact that NFs show a Newtonian behavior [38, 39], while many other non-Newtonian NFs show mainly shear-thinning behavior [40, 41]. Some articles reported that the *μ*_*nf*_ decreases with increasing NP size [42, 43]. The results show that the *μ*_*nf*_ decreases with increasing temperature [35, 44]. Today, liquids (for coolants) can be used to reduce friction between different parts of moving engine. For example, the use of engine oil increases efficiency and reduces fuel consumption. Oil is one of the most widely used fluids in various components of engines, which has many uses for cooling (See Figure 1).

One of the factors that lead to energy loss, increased pollution and fuel consumption in cars is the caused friction by the movement of components in the car engine. Using good engine oils can solve these problems. Also, using NPs in engine oil can eliminate these problems. The use of these properties in engine oil leads to improving the lubrication process and repairing worn surfaces and increasing engine power and torque, reducing pollution and gases and saving fuel consumption, which is one of the most important issues in the world. This technology was introduced in various fields of science and different industries; products were created based on this. Meanwhile, engine oil and fuel additives were also influenced by nanotechnology and related products have entered the market. Esfe et al. is one of the prioneer groups in the development of hybrid nanomaterials and nanofluid [45, 46]. The mentioned research group is one of the main research groups of NP sciences, Mono nanofluid and specially hybrid nanofluid. By introducing hybrid nanomaterials, they created a balance between the cost and performance of synthesized nanomaterials. They also began new research to control the *μ*_*nf*_ after the dispersion of NPs in the base fluid. the rheological behavior of CuO-MWCNT (85%–15%)/10W-40 HNF in the temperature range of T = 5–55 °C and SVF = 0.05–1% were investigated by Hemmat Esfe et al. [47]. The behavior of HNF was determined using the Herschel-Bulkeley (Bingham type) model, so that at T < 45 °C, the HNF shows non-Newtonian pseudo-Bingham behavior. In another study by Hemmat Esfe et al. [48], the dynamic viscosity of MWCNT (40%)-SiO_2_(60%)/5W50 HNF was experimentally measured in T = 5–55 °C, SVFs between 0 and 1% and SRs from 50 to 800 rpm. Investigation of the rheological behavior of HNF against shear stress shows that HNF has non-Newtonian behavior. Two methods of artificial neural network (ANN) and mathematical correlation were used to present the relationship between dynamic viscosity and independent parameters. The results show that the proposed correlation can estimate the value of dynamic viscosity with acceptable accuracy. The purpose of this research is to add suitable NPs to the engine oil that has a suitable viscosity in different atmospheric conditions. HNFs with these characteristics will increase the efficiency and quality of the produced engine oil and prevent possible damage to the moving parts of the engine. It slows down and increases the life of the engine, which will be of interest to the craftsmen. They found that at all temperatures and SVFs, the *μ*_*nf*_ was dependent on SR, and the NFs exhibit non-Newtonian shear-thinning behavior. Also, their analytical results show that the *μ*_*nf*_ decreases and increases with increasing temperature and SVF, respectively.

In this study, laboratory, statistical and numerical analyzes with different objectives were used to investigate the rheological behavior of MWCNT (25%)-MgO (75%)/SAE40 HNF. In the laboratory study, the effect of temperature, SVF and SR on μ_nf_ will be investigated. Then, by choosing the appropriate model, two correlations are proposed for the viscosity and prediction of nanofluid so that it can be used for numerical methods. In the final part, it was modeled and predicted by RSM.

## 2 Laboratory: equipment and measurements

**Figure 2:**
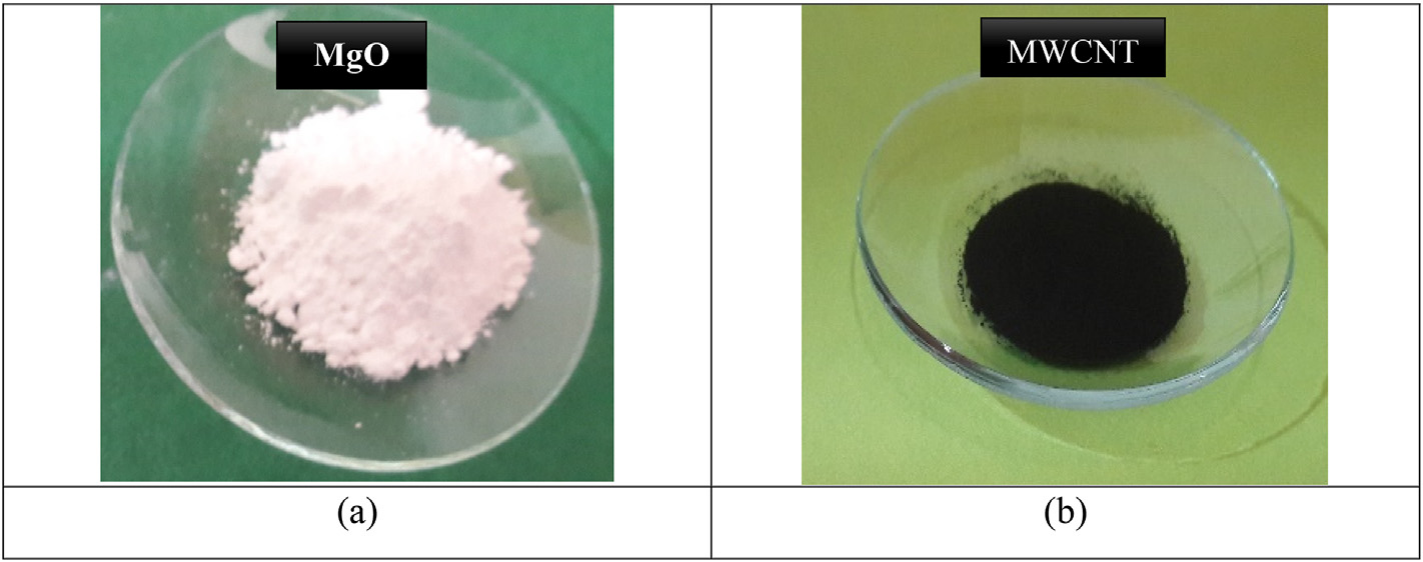
MgO and MWCNT NPs.

In this research, NPs were prepared in nano dimensions during a material engineering process and in a common two-step method (Figure 2). The first part of preparing NFs is the dispersion of NPs in the base fluid is then stabilizing the suspension. In this work, SAE40 oil was used as the base fluid. Additive NPs consist of MWCNT and MgO NPs with a composition ratio of 25%: 75%. Figure 2a shows MgO NPs and Figure 2b shows MWCNT NPs.

**Figure 3:**
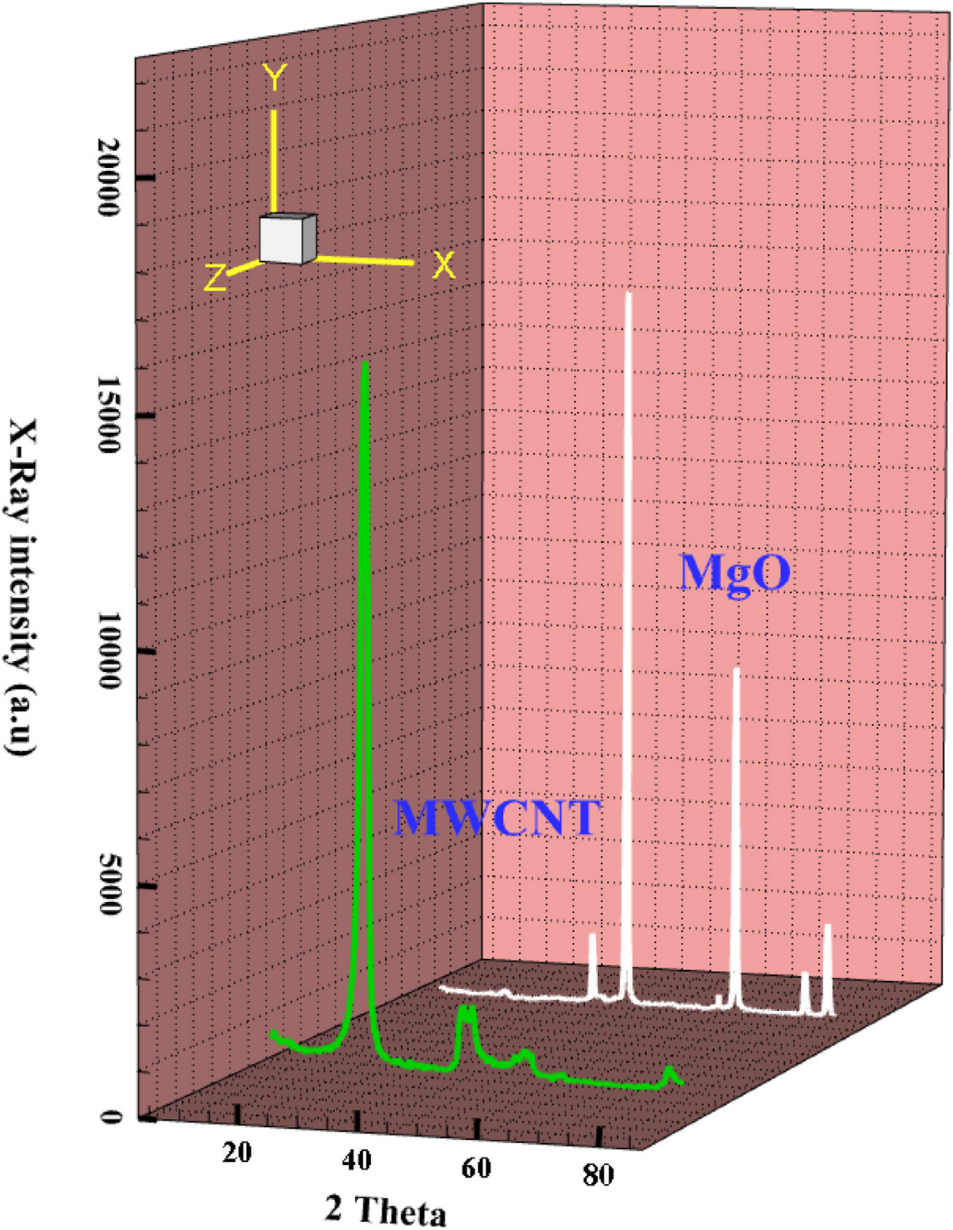
XRD analysis of NPs.

The structural and morphological properties of MgO and MWCNTs were measured using high magnification X-ray diffraction (XRD) as shown in Figure 3

**Figure 4:**
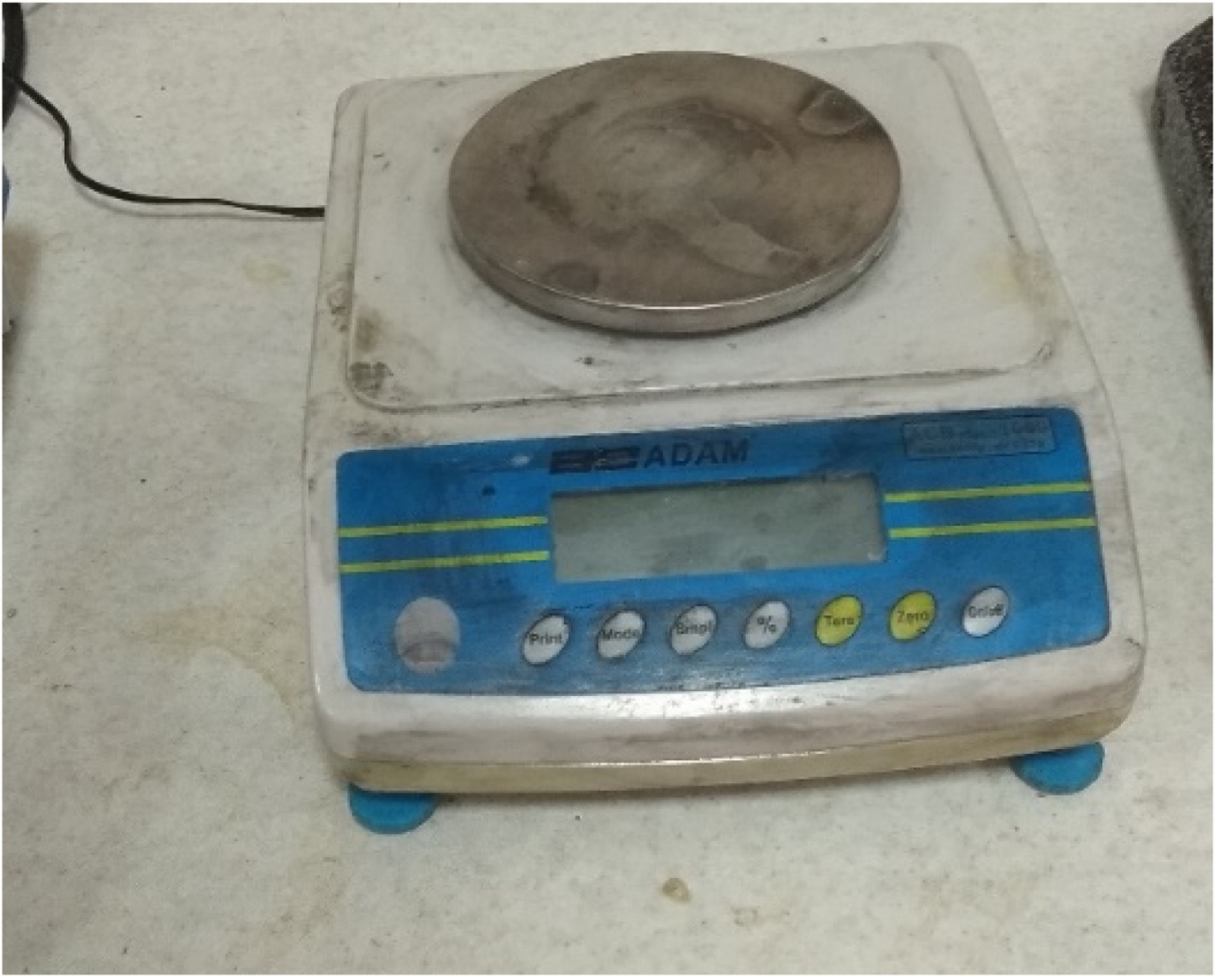
Digital scale for weighing NPs.

The amount of required MWCNT and MgO NPs for different SVFs can be determined using Eq. (1). The NP samples were weighed using a sensitive electronic scale with an accuracy of 1 mg Figure 4.

(1) $$SVF=\frac{{\frac{w}{\rho }|}_{MWCNT}+{\frac{w}{\rho }|}_{MgO}}{{\frac{w}{\rho }|}_{MWCNT}+{\frac{w}{\rho }|}_{MgO}+{\frac{w}{\rho }|}_{SAE40}}\times 100$$

**Figure 5:**
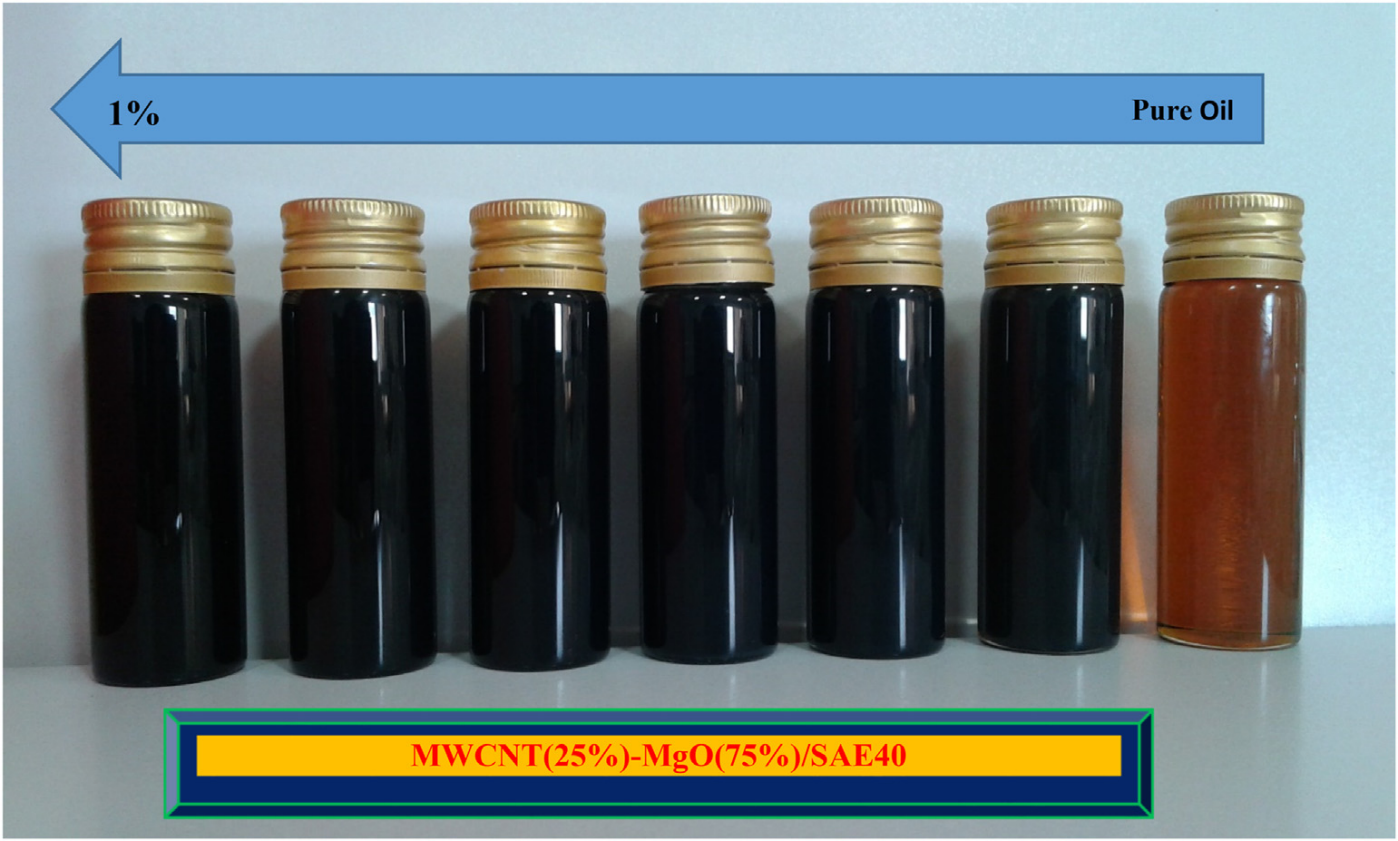
Stability of HNFs at different SVFs.

After weighing the NPs, a magnetic stirrer and ultraonic vibration were used to disperse and suspend the NPs in the base oil. This results in a stable suspension and uniform dispersion. The nanofluid with good dispersion and stability after 25 days were prepared to investigate the rheological behavior.

**Figure 6:**
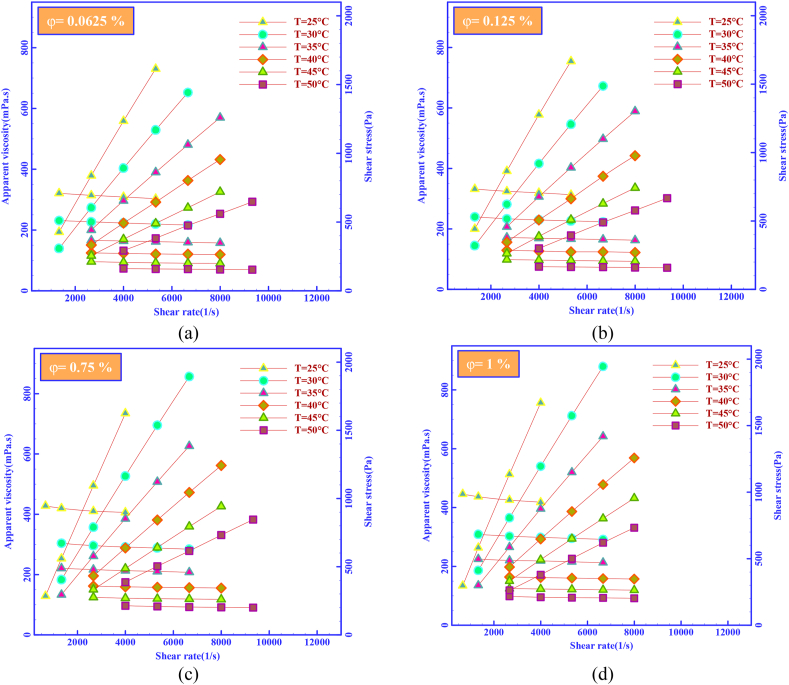
Flow curve at different temperatures.

Figure 5 shows the stabilized HNFs in different SVFs. More information about the properties of MgO and MWCNT NPs is presented in Table 1.

**Figure 7:**
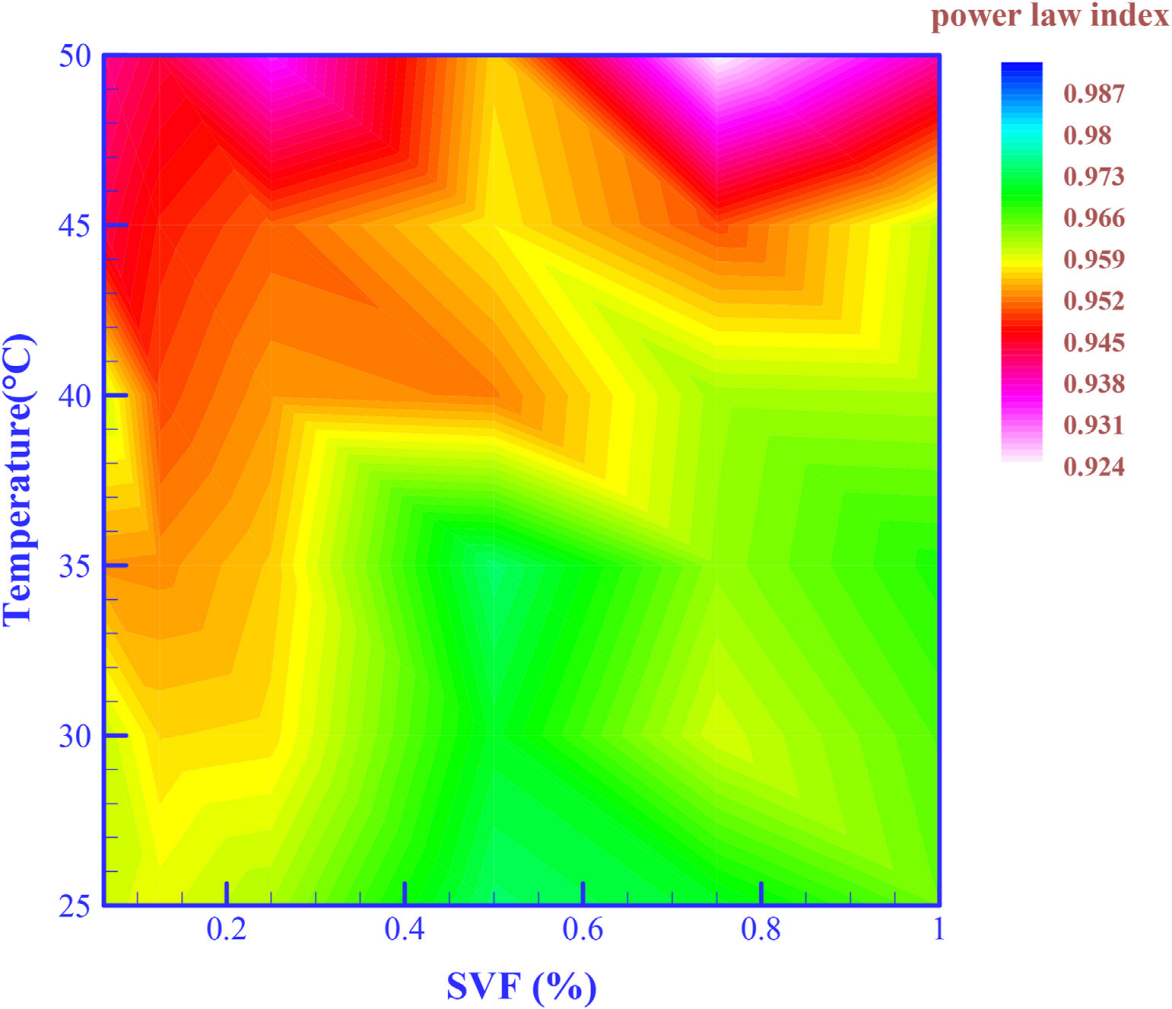
The contour of the power-law index.

**Table 1:** Properties of MgO and MWCNT NPs.

Specifications	NPs	
	MWCNTs	**MgO**
Purity	>95 wt%	99+%
Color	Black	white
Morphology	Cylindrical	polyhedral

### 2.1 Viscosity measurement

To analyze the rheological behavior of HNFs, the viscosity of MgO-MWCNTs/SAE40 HNF with different SVFs (0.0625%, 0.125%, 0.25%, 0.5%, 0.75% and 1%) at T = 25–50 °C was measured by Brookfield CAP2000 viscometer. This device is provided by the Brookfield Engineering Laboratory in America. Before starting to work, the device was calibrated and its error was measured. The viscometer was tested with SAE40 oil at T = 25 °C before measuring the μ_nf_. Table 2 gives the range of conditions for measuring the μ_nf_. To increase the accuracy of the measurements and as a result, provide a more accurate analysis, all the experimental data were repeated at most five times and then their average was recorded. Some measured data are reported in Table 3.

**Table 2:** Range of conditions for measuring the *μ*_*nf*_

HNF	Range of laboratory conditions		
	T (°C)	SVF (%)	SR (s^−1^)
MWCNT-MgO(25:75)/SAE40	25–50	0.0625–1	666.5–7998

**Table 3:** Some measured data by a CAP2000 + viscometer.

HNF	SVF (%)	T (°C)	SR (s^−1^)	***μ***_***nf***_ (mPa.s)
MWCNT-MgO(25:75)/SAE40	0.0625	25	1333	321
	0.125	30	2666	233.4
	0.25	35	3999	188.1
	0.5	40	5332	154.7
	0.75	45	6665	119.2
	1	50	7998	91.6

## 3 Discussion

### 3.1 Rheological behavior

#### 3.1.1 Effect of **SR**

In the first step of this study, the rheological behavior of the HNF in Newtonian and non-Newtonian groups was investigated in three-dimensional curves. The τ→SR curve is generally called the flow curve, where the shear stress (τ) is a force that can create continuous and constant deformation in the fluid. Unlike non-Newtonian fluids, Newtonian fluids are fluids in which the ratio of shear stress and SR is linear, and a non-Newtonian fluid is a fluid whose viscosity changes with the applied SR. At the beginning of this study, to determine the flow behavior of HNFs, their rheograms at different temperatures and SVFs are examined and compared. Using rheological curves and matching it with μ_nf_ of MWCNT-MgO (25:75)/SAE40 HNF, the flow behavior of HNF was investigated. In the curves of Figure 6 (a-d), the basis of the changes is SR. Hence, Figure 6 (a) is for SVF = 0.0625%, Figure 6 (b) is for SVF = 0.125%, Figure 6 (c) is for SVF = 0.75% and Figure 6 (d) is for SVF = 1%. According to these figures, μ_nf_ changes at the studied temperatures (T = 25–50 °C) with changes in SR. The pattern of studied HNF flow behavior corresponds to the non-Newtonian fluids.

**Figure 8:**
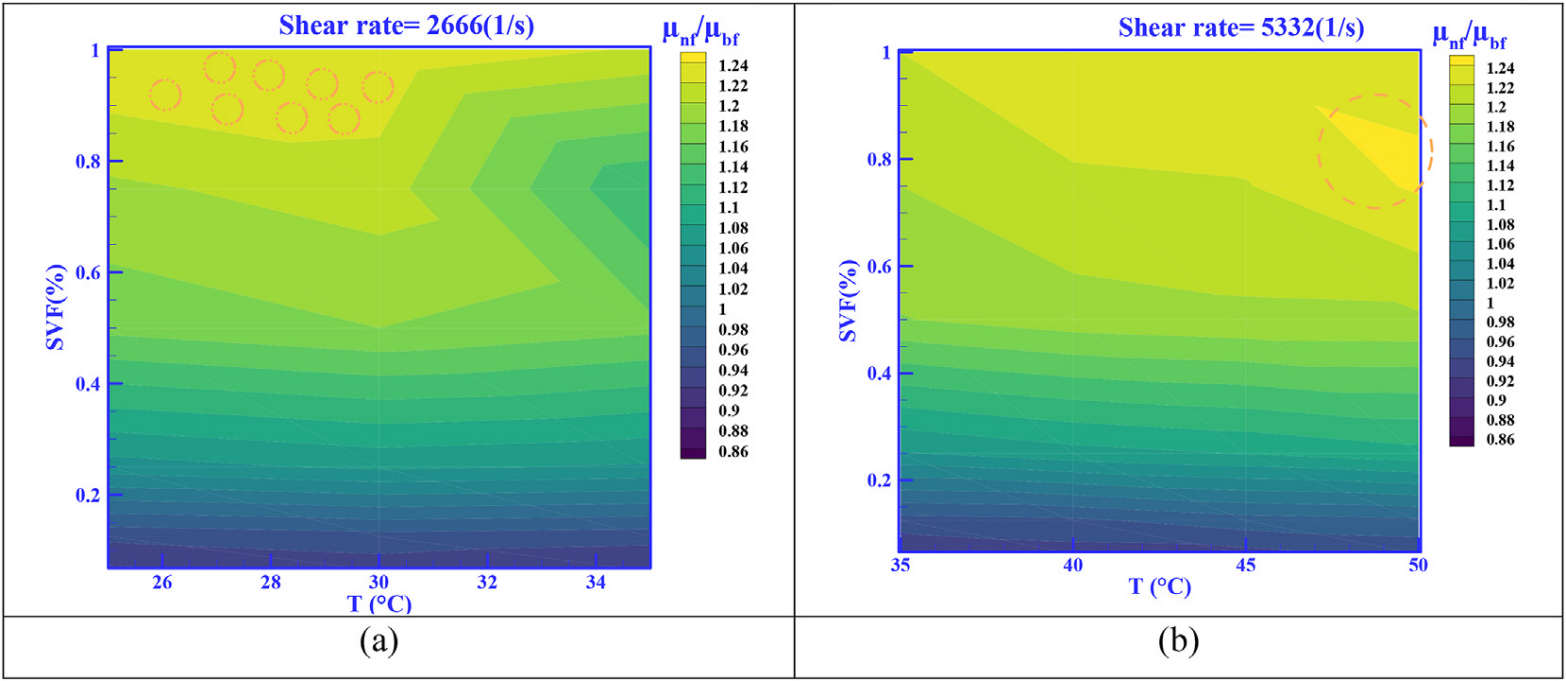
μ_r_ contour at SR = 2666 and 5332 s^−1^

#### 3.1.2 Power-law index (n)

Following the path of detecting HNF flow behavior, the power-law contour is shown in Figure 7. Eq. (2) is used to identify the rheological behavior of HNFs. According to the contour of Figure 7, it can be seen that the power-law index is in the range 1 > n > 0. The mentioned values confirm the behavior mentioned in the previous section (effect of parameter SR).

(2) $$\tau =m{*SR}^{n}$$

**Figure 9:**
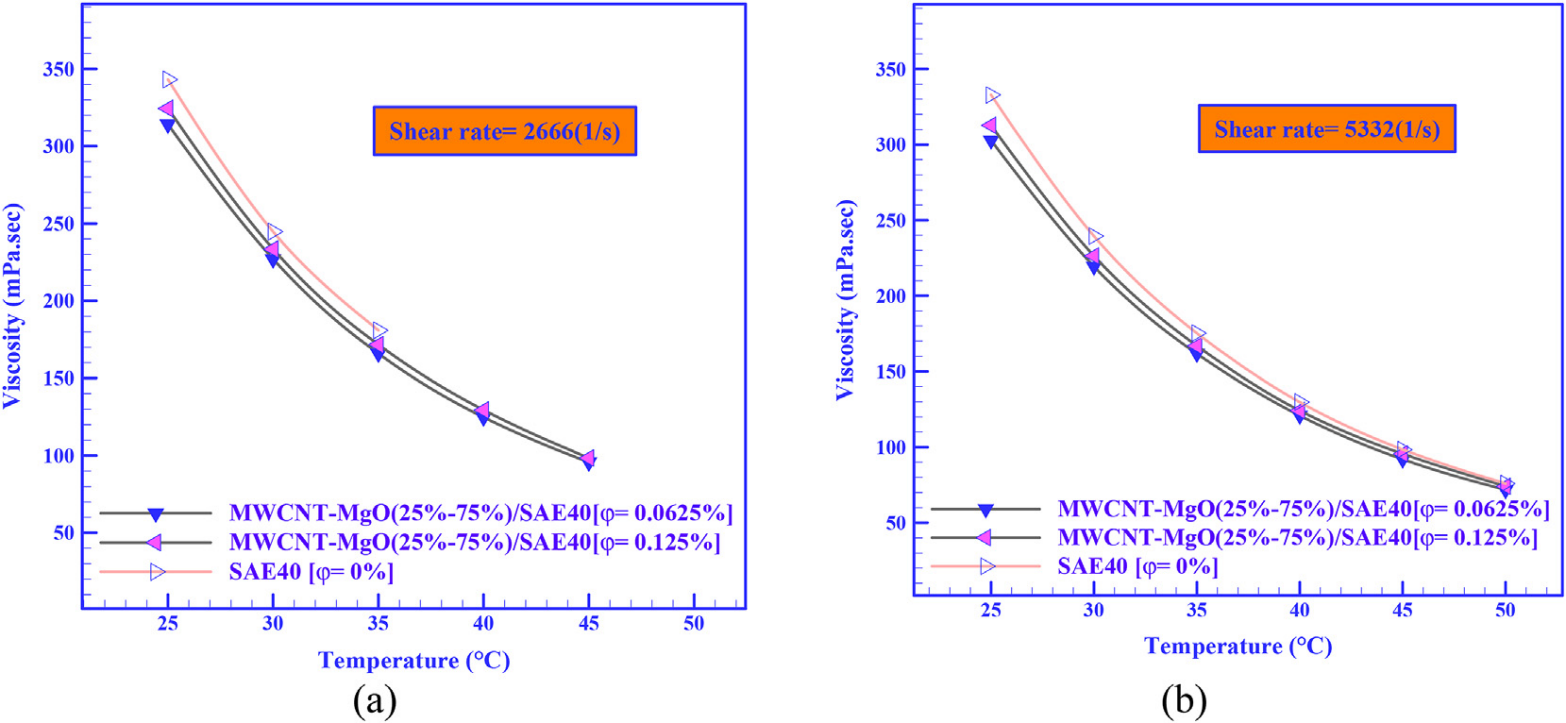
Difference between *μ*_*nf*_ and *μ*_*bf*_ at SR = 2666 and 5332 s^−1^ and different SVFs.

Numerical values of the power-law index are reported in Table 4. HNF [MWCNT-MgO (25:75)/SAE40] at T = 50 °C and SVF = 0.75% has the strongest non-Newtonian behavior and n = 0.9233.

**Table 4:** Power-law index values at different SVFs and different temperatures.

HNF		Power-law index (n)					
		**T** = 25 °C	**T** = 30 °C	**T** = 35 °C	**T** = 40 °C	**T** = 45 °C	**T = 50°C**
MWCNT-MgO (25:75)/SAE40	**SVF** = 0.0625%	0.9593	0.9599	0.9528	0.9597	0.9431	**0.9593**
	SVF = 0.125%	0.9586	0.9559	0.9525	0.9493	0.9472	0.9433
	SVF = 0.25%	0.9611	0.9564	0.9552	0.9528	0.9503	0.9334
	SVF = 0.5%	0.9734	0.9704	0.9736	0.9517	0.957	0.9556
	SVF = 0.75%	0.9705	0.9592	0.9623	0.9616	0.9494	0.9233
	SVF = 1%	0.964	0.9652	0.9676	0.9612	0.9604	0.9384

### 3.2 Viscosity comparison

#### **3.2.1** Relative **μ**_**nf**_ (**μ**_**r**_)

To better understand the viscosity behavior, the μ_r_ contour at SR = 2666 (See Figure 8 a) and 5332 s^−1^ (See Figure 8 b) is shown in Figure 8 (a and b). μ_r_ is calculated using Eq. (3).

(3) $$\mu_{r\;}=\frac{\mu_{nf}}{\mu_{bf}}$$

**Figure 10:**
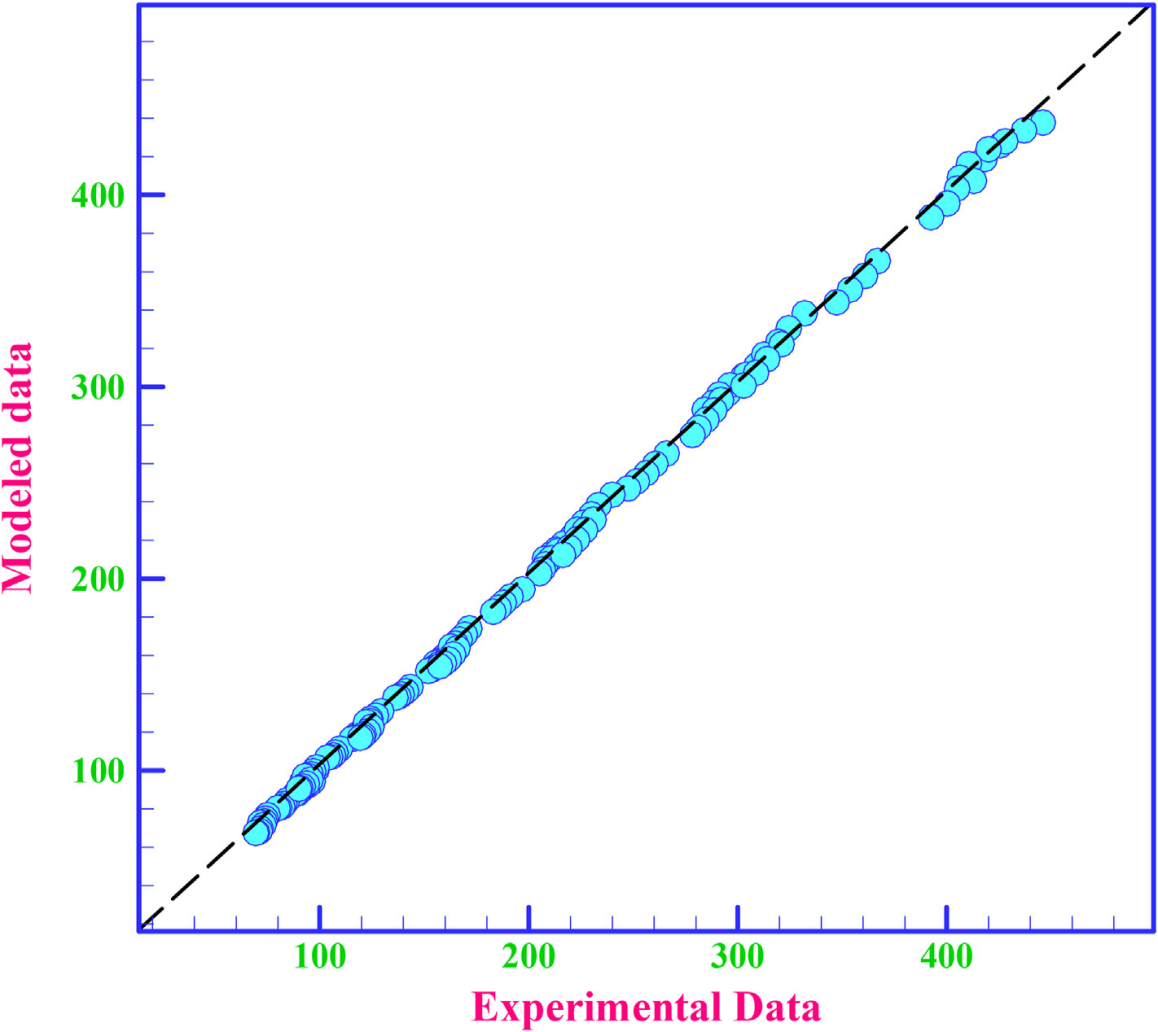
Correlation of modeled data with experimental data.

In Figure 8 (a) and at SR = 2666 s^−1^, the largest increase in μ_r_ was 1.24, which is equal to +24%. The numerical results of μ_r_ are reported in Table 5.

**Table 5:** Statistical data on the percentage of μ_r_ changes.

**HNF**	***SR* (1/s)**	**T (°C)**	μ_r_			
			**SVF**=0.0625%	**SVF**=0.125%	**SVF**=0.75%	**SVF**=1%
MWCNT-MgO (25:75)/SAE40	2666 (200rpm)	T = 25	0.915 (-8.50%)	0.945	1.196	1.24 (+24%)
		T = 30	0.927	0.953	1.21	1.237
		T = 35	0.917	0.948	1.197	1.217
	5332 (400rpm)	T = 35	0.922	0.952	1.20	1.22
		T = 40	0.931	0.956	1.21	1.234
		T = 45	0.933	0.971	1.22	1.238
		T = 50	0.944	0.976	1.231	1.235

#### **3.2.2** μ_nf_**→T curve**

Among other presented comparisons in this scenario, which is very important in determining the optimal HNF, is the examination of viscosity curves in terms of temperature. The most important and effective parameter in this field is temperature. One of the important goals is to compare the difference in the viscosity of HNFs compared to the base fluid at different temperatures. This comparison will ultimately lead to the selection of the most optimal mode in the preparation of HNF. Therefore, for example, in Figure 9 (a and b), the viscosity curves based on the temperature at the highest and lowest SRs (SR = 2666 and 5332 s^−1^) and SVFs were compared. Figure 9a is for SR = 2666 s^−1^ and Figure 9b is for SR = 5332 s^−1^. According to Figure 9, a drop-in viscosity has been seen for both HNFs at all temperatures, but the viscosity is not much different from the base oil.

A comparison of the difference between μ_nf_ and μ_bf_ in SR = 2666 and 5332 s^−1^ and SVF = 0.0625 % and 0.125% is reported in Table 6. According to Table 6, at SR = 2666 s^−1^ and SVF = 0.0625 % at T = 25 °C, the HNF has the lowest viscosity percentage of -8.45%.

**Table 6:** A comparison of the difference between the *μ*_*nf*_ and the *μ*_*bf*_ at SR = 2666 and 5332 s^−1^ and SVF = 0.0625% and 0.125%.

SR (s^−1^)	T (°C)	$\Delta {\left(\mu_{n-b}\right)}_{f}|SVF=0.0625\;\%$	$\Delta {\left(\mu_{n-b}\right)}_{f}|SVF=0.125\;\%$
		MWCNT-MgO (25:75)/SAE40	MWCNT-MgO (25:75)/SAE40
2666	25 °C	-29.00 (-8.45%)	-18.70
	30 °C	-17.80	-11.30
	35 °C	-15.00	-9.30
5332	40 °C	-8.90	-5.60
	45 °C	-6.50	-2.80
	50 °C	-4.20	-1.80

## 4 Impractical results

### 4.1 Correlation model of the RSM

RSM is one of the important procedures in examining the target response to achieve the relationship between the independent variable and the dependent variable. The RSM was used for dependent variable analysis where one or more dependent variables (as a response) were affected by many variables and the objective is to optimize the response. In RSM, the governing equation for the rheological behavior of HNFs and the effective parameters and interactions are determined, and finally, the target response can be optimized based on the effective parameters. In this research, the optimization process was carried out using the three-variable model with the coefficient of determination of R^2^ = 0.9994, and then a non-linear mathematical relationship with the parameters of temperature (T), SVF and SR were presented to predict the experimental data (Eq. 4). In Tables 7 and 8, the related values to the parameters affecting the performance of the modeler and the accuracy of the equation are reported.

(4) μ_nf_ = +1439.18463–74.55874T + 680.60734*SVF*-0.023008*SR*-18.93092T*∗SVF* +9.20862E-004T*∗SR*+1.41458T^2^-335.84851*SVF*^2^+1.64364E-007*SR*^2^+0.14964T^2^*SVF*-1.03952E-005 T^2^∗*SR*+3.83013 T*∗SVF*^2^-9.38513E-003 T^3^ + 68.56743*SVF*^3^

**Table 7:** ANOVA for Response Surface Reduced Cubic model.

ANOVA table [Partial sum of squares - Type III]						
Source	**Sum of Squares**	**df**	**Mean Square**	**F-Value**	**P-value**	
					**Prob > F**	
Model	1.684E+006	13	1.295E+005	22252.48	<0.0001	significant
A-T	1.297E+005	1	1.297E+005	22279.76	<0.0001	
B-SVF	2035.45	1	2035.45	349.69	<0.0001	
C-SR	669.38	1	669.38	115.00	<0.0001	
AB	17589.04	1	17589.04	3021.76	<0.0001	
AC	319.22	1	319.22	54.84	<0.0001	
A^2^	62812.91	1	62812.91	10791.11	<0.0001	
B^2^	8369.61	1	8369.61	1437.88	<0.0001	
C^2^	49.06	1	49.06	8.43	0.0042	
A^2^B	1603.49	1	1603.49	275.48	<0.0001	
A^2^C	211.61	1	211.61	36.35	<0.0001	
AB^2^	1247.53	1	1247.53	214.32	<0.0001	
A^3^	1912.26	1	1912.26	328.52	<0.0001	
B^3^	322.94	1	322.94	55.48	<0.0001	
Residual	931.33	160	5.82			
Cor Total	1.685E+006	173				

In this test, the R^2^ is equal to 0.9999 and the P-value is less than 0.0001, which indicates the appropriateness of the proposed correlation.

**Table 8:** Values of measurements accuracy of regression analysis.

Std. Dev.	2.41	R-Squared	0.9994
Mean	188.50	Adj R-Squared	0.9994
C.V. %	1.28	Pred R-Squared	0.9993
PRESS	1139.49	Adeq Precision	541.007
-2 Log Likelihood	785.69	BIC	857.91

To check the appropriateness and significance of the model, Fisher's test parameters (F-test), probability (P-value) and coefficient of variation (R-square) were used in the analysis of variance (ANOVA).

As can be seen in Figure 10, the modeled data provide good agreement and correlation with the corresponding empirical data, which indicates the acceptable validity of the proposed relationship.

## 5 Conclusion

In this study, the rheological behavior of MWCNT-MgO(25%:75%)/SAE40 HNF as laboratory and statistical analysis with different objectives at T = 25–50 °C and SVF = 0.0625, 0.125, 0.25, 0.5, 0.75 and 1% were measured and reported. The results show that traditional models are not suitable for predicting the viscosity of MWCNT-MgO/SAE40 HNFs. The main results of this study are classified as follows:

• Temperature and SVF are two important and effective parameters of viscosity. The effect of temperature on viscosity is superior to the effect of SVF on viscosity.
• It was found that the relationship between shear stress and SR is nonlinear and viscosity has non-Newtonian behavior.
• An increase in temperature weakens van der Waals forces. Therefore, an increase in temperature led to a decrease in viscosity. At T = 25 °C and SR = 2666 s^−1^, the viscosity has the lowest percentage and is equal to -8.45%.
• The statistical results of the laboratory study show that the maximum μ_nf_ and minimum μ_nf_ increase and decrease by -8.50% and 24%, respectively, compared to the base oil.
• Using RSM, a non-linear relationship with acceptable accuracy and quality with R^2^ = 0.9994 was presented to predict the experimental data and establish the relationship between the response function and the independent variables.
• The studied HNFs with these characteristics will increase the efficiency and quality of the produced engine oil and prevent possible damage to the moving parts of the engine. It reduces the speed and increases the life of the engine, which will be of interest to the craftsmen.

## Declarations

### Author contribution statement

Mohammad Hemmat Esfe, Davood Toghraie, Soheyl Alidoust, Fatemeh Amoozadkhalili, Erfan Mohammadnejad Ardeshiri: Conceived and designed the experiments; Performed the experiments; Analyzed and interpreted the data; Contributed reagents, materials, analysis tools or data; Wrote the paper.

### Funding statement

This research did not receive any specific grant from funding agencies in the public, commercial, or not-for-profit sectors.

### Data availability statement

No data was used for the research described in the article.

### Declaration of interests statement

The authors declare no conflict of interest.

### Additional information

No additional information is available for this paper.
